# Supplementary material for: Characterization of a C-Type Lectin Domain-Containing Protein with Antibacterial Activity from Pacific Abalone (Haliotis discus hannai)
Source: Int J Mol Sci. 2022 Jan 9;23(2):698. doi: 10.3390/ijms23020698 (PMC8775961; doi:10.3390/ijms23020698)
Supplement: Supplementary file 1 [file ijms-23-00698-s001.zip › ijms-1488530-supplementary.pdf]

Table S1. List of protein sequences used in phylogenetic tree construction.

Name in Phylogenetic tree	Organism	Definition	Accession
H. laevigata Perlucin	*Haliotis laevigata* (Greenlip abalone)	Perlucin	P82596
H. ddiscus Perlucin 1	*Haliotis discus discus* (Disc abalone)	perlucin 1	ABO26590.1
M. galloprovincialis CTL6	*Mytilus galloprovincialis* (Mediterranean mussel)	C-type lectin 6	AJQ21497.1
A. irradians CTL	*Argopecten irradians* (Bay scallop)	C-type lectin	ADL27440.1
P. flavescens CTL3	*Perca flavescens* (Yellow perch)	C-type lectin 3	ACO82036.1
M. galloprovincialis CTL2	*Mytilus galloprovincialis* (Mediterranean mussel)	C-type lectin 2	AJQ21493.1
M. galloprovincialis CTL3	*Mytilus galloprovincialis* (Mediterranean mussel)	C-type lectin 3	AJQ21494.1
H. diversicolor Perlucin 5	*Haliotis diversicolor*	perlucin 5	AEQ16379.1
C. virginica CTLD10	*Crassostrea virginica* (Eastern oyster)	C-type lectin domain family 10 member A-like isoform X1	XP_022293075.1
R. philippinarum CTL	*Ruditapes philippinarum* (Manila clam)	C-type lectin domain family member isoform crab 3	AZS54110.1
M. musculus Clec10A	*Mus musculus* (Mouse)	C-type lectin domain family 10 member A	P49300
M. musculus Collectin	*Mus musculus* (Mouse)	Collectin-12	Q8K4Q8
L. unguis Perlucin	*Lingula unguis*	Perlucin	A0A1S3IWF5
P. reticulata CTLD protein	*Poecilia reticulata* (Guppy)	C-type lectin domain-containing protein	A0A3P9N573
P. nyererei CTLD protein	*Pundamilia nyererei*	C-type lectin domain-containing protein	A0A3B4F105
O. niloticus CTLD protein	*Oreochromis niloticus* (Nile tilapia)	C-type lectin domain-containing protein	I3JBE6
M. yessoensis Perlucin-like protein	*Mizuhopecten yessoensis* (Japanese scallop)	Perlucin-like protein	A0A210R405
M. yessoensis Perlucin	*Mizuhopecten yessoensis* (Japanese scallop)	Perlucin	A0A210Q3W2
C. gigas Perlucin	*Crassostrea gigas* (Pacific oyster)	Perlucin	K1QRE6
N. brichardi CTLD protein	*Neolamprologus brichardi* (Fairy cichlid)	C-type lectin domain-containing protein	A0A3Q4MIS0
C. gigas CTMR2	*Crassostrea gigas* (Pacific oyster)	C-type mannose receptor 2	K1PCU5

Abbreviations: CTL, C-type lectin; CTLD, C-type lectin domain-containing protein.
